# Rising complexity of the OAM beam structure as a way to a higher data capacity

**DOI:** 10.1038/s41377-022-00909-2

**Published:** 2022-07-13

**Authors:** Andrey Pryamikov

**Affiliations:** Prokhorov General Physics Institute of the Russian Academy of Sciences, Moscow, Russia

**Keywords:** Fibre optics and optical communications, Optoelectronic devices and components

## Abstract

Standard vortex beams carrying different OAM (optical angular momentum) modes can provide independent communication channels for information transmission. However, they are unable to reach the capacity limit of a communication channel due to a rapid divergence of the beams with high values of the OAM order. The solution can be found by using multi-vortex geometric beams.

The electromagnetic waves are known to carry angular momentum. The angular momentum itself can be decomposed into a spin component (the photon spin is manifested in circular polarization) and an orbital component that can be significantly greater than the spin in the case of the optical vortex beams^1^. Vortex beams are characterized by Hilbert factor exp(*il*ϕ), where ϕ is an azimuthal angle and *l* is an integer number that can be quite large (OAM is equivalent to $\hbar$*l* per photon). Beams with different OAM values can be orthogonal to each other and can be considered as a subset of the Laguerre–Gaussain (LG_*lp*_) modal basis set. The OAM mode with a nonzero order (*l* ≠ 0) has a donut shape intensity profile and helical phase front. The size of the ring intensity profile grows with *l* while *p* characterizes the number of concentric amplitude rings (with one ring at *p* = 0) (Fig. 1(left)). The orthogonality of the Laguerre–Gaussain modes allows multiplexing and de-multiplexing of independent optical beams with a minimal inherent cross-talk^2,3^. Thus, the OAM of light can be used as an independent optical degree of freedom for multiplexing modulation to enlarge the capacity of optical communications.

**Fig. 1:**
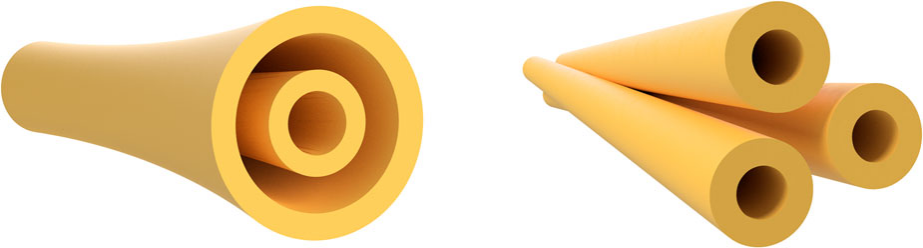
Two types of modes. A schematic diagram of LG beam (left) and MVGB (right)

Currently, there are two areas of development of optical communications using OAM modes. The first method is OAM shift keying when the information is encoded in the *l* value of the OAM beam. The second method that is important to us is OAM division multiplexing when the information is multiplexed in multimodal OAM beams. In this case, the data is loaded into different OAM modes, multiplexed, and transmitted through a single aperture. The beams are collected at the receiver, demultiplexed, and detected for data recovery. OAM division multiplexing is a subset of space division multiplexing^4^ and can be compatible with different modulation formats. Besides, OAM space division multiplexing has a higher spectral efficiency and exhibits lower bit error rates in comparison with OAM shift keying. The use of OAM multiplexing made it possible to raise the communication capacity to the Tbit level^3,5^.

In the case of free-space, OAM multiplexed optical communication links, there are several problems leading to intermodal power coupling and deleterious inherent cross-talk^6^. One of these problems is related to divergence when free-space beams of higher OAM orders diverge faster than lower-order beams. It is obvious that the divergence makes it difficult to fully capture the higher-order OAM beam at a limited-sized receiver aperture leading to an increased power loss. This, in turn, gives rise to the capacity limit of a communication channel^7^. In addition, modal coupling can occur due to the truncation of a beam’s radial profile^6^.

To solve this problem, writing in this issue of *Light: Science & Applications*, Zhensong Wan and colleagues at Key Laboratory of Photonic Control Technology (Tsinghua University), State Key Laboratory of Precision Measurement Technology and Instruments (Tsinghua University) and Optoelectronics Research Center (University of Southampton) suggested using a set of multi-vortex geometric beams (MVGBs) as high-dimensional information carriers^8^ (Fig. 1(right)). In the case of MVGBs, it is possible to obtain three additional independent degrees of freedom including central OAM, sub-beam OAM, and coherent-state phase. The beams with multi-singularity optical fields have found special applications in multiple particle manipulation^9^, 3D displays^10^, and optical modulation^11^.

The authors have demonstrated that the modal basis of MVGBs outperforms the OAM and LG modal basis in terms of approaching the capacity limit of a communication channel. They created a three-dimensional set of orthogonal data-carrying beams by using three independent intrinsic degrees of freedom of the above MVGBs. It was shown that a MVGB set has a divergence variation of 18% among 100 independent lowest order spatially multiplexed modes. The divergence variation for an OAM set is 900% and for a LG set is 429%, respectively. It means that thousands of independent spatial channels in the MVGB basis can be supported by free-space communication links. It is two orders of magnitude larger than that in OAM basis. Moreover, the authors carried out proof-of-concept experiments of the tri-degrees of freedom mode multiplexing–demultiplexing and shift-keying encoding/decoding. Specifically, a successful data-packet transmission with a much lower pixel error rate than in OAM beams was demonstrated.

The authors point out that MVGB multiplexing can be combined with wavelength and polarization division multiplexing which can help in the implementation of the next-generation high-capacity free-space optical communication links. Also, the concept of tri-degrees of freedom can be applied to encoding and decoding in the quantum data channels.
